# The interplay between childhood obesity and vitamin D deficiency: mechanisms and implications

**DOI:** 10.3389/fped.2025.1700949

**Published:** 2026-01-13

**Authors:** Xiao Zhou, Yuhe Chen, Jianxin Xu, Xiaobing Li, Yanmei Wu, Lidan Xu

**Affiliations:** 1 Department of Pediatrics, Jinhua Municipal Central Hospital, Jinhua, Zhejiang, China; 2 Department of Pediatrics, Jinhua Maternal and Child Health Care Hospital, Jinhua, Zhejiang, China; 3 Department of Pediatrics, Sichuan Provincial People’s Hospital, School of Medicine, University of Electronic Science and Technology of China, Chengdu, Sichuan, China

**Keywords:** childhood obesity, obesity management, vitamin D deficiency, vitamin D signaling, vitamin D supplementation

## Abstract

Childhood obesity and vitamin D deficiency (VDD) are intertwined global health threats that amplify each other beyond their individual skeletal or metabolic consequences. This review synthesises a decade of clinical, translational and Mendelian-randomisation evidence to show that obesity drives VDD through volume dilution, adipose-tissue sequestration and metabolic associated steatotic liver disease (MASLD)-related hydroxylase dysfunction, while VDD in turn aggravates adipogenesis, leptin resistance and chronic low-grade inflammation, locking children into a self-perpetuating cycle. Meta-analysis of 12 randomised trials (*n* = 1,538) revealed that vitamin D supplementation improves insulin sensitivity and lowers systolic blood pressure only when baseline 25-hydroxyvitamin D (25(OH)D) is <20 ng/mL and systemic inflammation is modest; no consistent reduction in BMI or fat mass was observed. Obese youths require 2–3 times the standard dose to reach 30 ng/mL, yet incremental metabolic benefit plateaus near 4,000 IU/day and is lost when systemic inflammation is present. Precision-dosing algorithms incorporating VDR/CYP2R1/DBP genotypes and MASLD status are urgently needed. Multi-omic longitudinal trials should clarify the adipose-tissue threshold at which hepatic cytochrome P450 family 27 subfamily B member 1 (CYP27B1) becomes substrate-limited and evaluate the vascular safety of intermittent high-dose boluses before personalised supplementation is translated into routine paediatric practice.

## 1 Introduction

Childhood obesity has become a significant public health challenge worldwide, with its prevalence increasing dramatically over the past few decades (1). This alarming trend poses substantial threats to children's growth and development, as well as their long-term health. Obesity in childhood is associated with a range of adverse health outcomes, such as cardiovascular diseases, type 2 diabetes, metabolic associated steatotic liver disease (MASLD), and certain types of cancer (2). Moreover, children who are obese are more likely to become obese adults, thereby facing an increased risk of chronic diseases like cardiovascular disease, diabetes, and cancer in adulthood (3). Given these serious implications, the prevention and management of childhood obesity are of paramount importance to improve children's health and reduce the incidence of chronic diseases in adulthood.

Vitamin D, a fat-soluble vitamin, is primarily synthesized in the skin upon exposure to ultraviolet B radiation from sunlight and can also be obtained through dietary intake (4, 5). It plays a crucial role in various physiological functions, including the regulation of calcium and phosphorus metabolism, maintenance of bone health, modulation of the immune system, and involvement in cell differentiation and proliferation (6). In recent years, the prevalence of vitamin D deficiency (VDD) has been increasing globally, particularly among children and adolescents (7). VDD can lead to impaired bone development, weakened immune function, and an increased risk of chronic diseases (8). Additionally, VDD has been linked to several metabolic disorders, such as insulin resistance and inflammation, which may further impact children's health (9).

An increasing body of evidence suggests a close relationship between childhood obesity and VDD (10). Children with obesity tend to have lower vitamin D levels compared to their normal-weight counterparts, which may be attributed to multiple factors, such as reduced outdoor activity, inadequate dietary intake, and the excessive storage of vitamin D in adipose tissue (11). Moreover, VDD may exacerbate obesity through mechanisms involving calcium metabolism, insulin sensitivity, and inflammatory responses (12). Therefore, exploring the interplay between childhood obesity and VDD is essential for developing effective prevention and intervention strategies. This review aims to summarize the current research progress on the association between childhood obesity and VDD, analyze the potential underlying mechanisms, and discuss future research directions.

## 2 Mechanisms of the vitamin D signaling pathway

### 2.1 Vitamin D: metabolism and activation

Vitamin D is a vital fat-soluble vitamin that exists in two primary forms: vitamin D_2_ (ergocalciferol) and vitamin D_3_ (cholecalciferol) (13). Vitamin D_3_ is predominantly synthesized in the skin from 7-dehydrocholesterol upon exposure to ultraviolet B (UVB) radiation, while vitamin D_2_ is primarily derived from certain fungi and plants (14). Human skin can convert 7-dehydrocholesterol into vitamin D_3_ under UVB irradiation (15). Additionally, vitamin D_3_ can be obtained through the consumption of animal-derived foods or supplements, whereas vitamin D_2_ is mainly found in specific fungi and plants (16).

**Figure 1:**
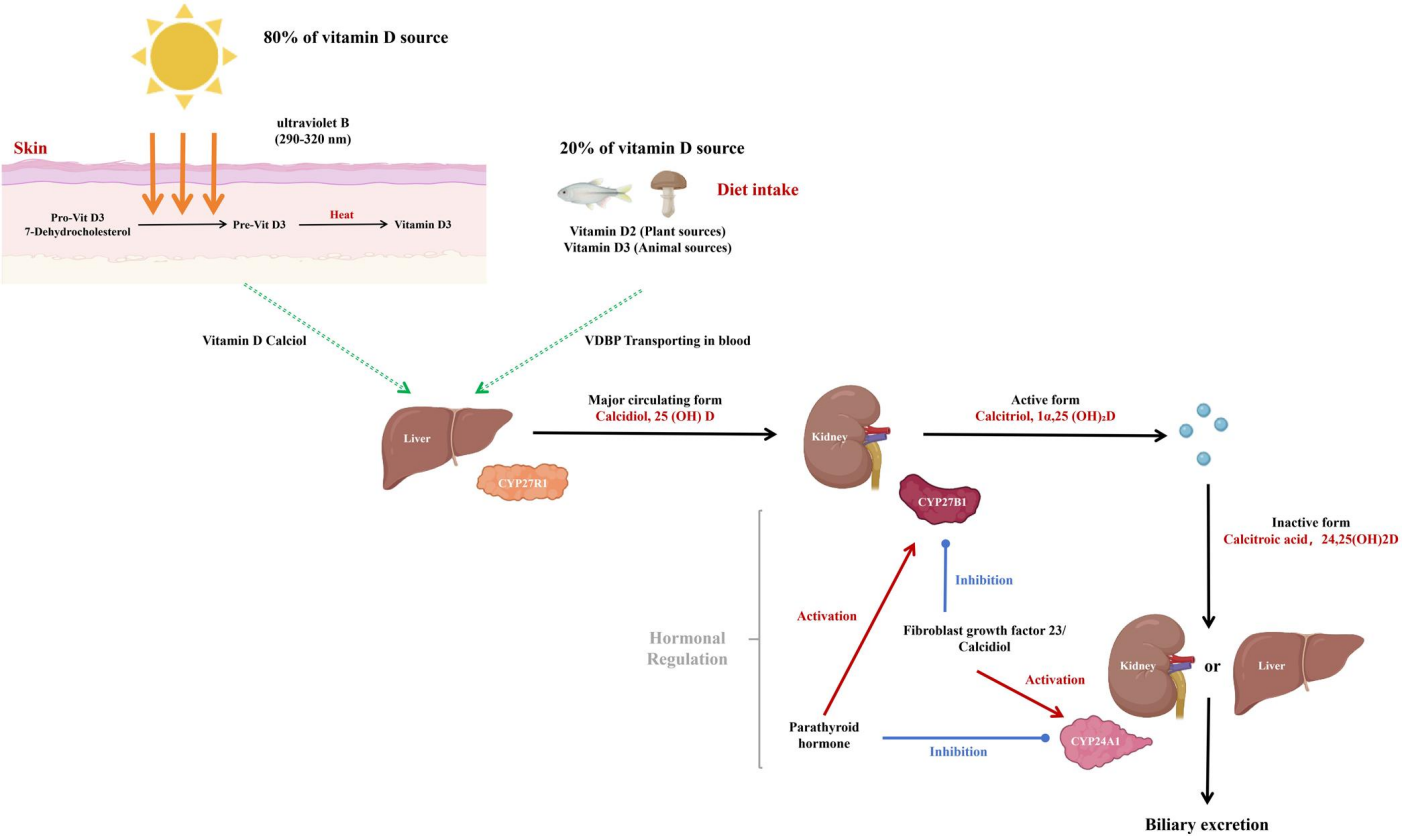
The metabolic and activation pathways of vitamin D3.

The metabolism of vitamin D involves two hydroxylation steps to convert it into its active form (see Figure 1). Initially, vitamin D is hydroxylated in the liver by the enzyme cytochrome P450 family 2 subfamily R member 1 (CYP2R1) to form 25-hydroxyvitamin D (25(OH)D), which is the principal circulating form of vitamin D (17).

The metabolism of vitamin D involves two sequential hydroxylation steps to convert it into its active form—a pathway first elucidated by DeLuca et al. (18) (see Figure 1). Initially, vitamin D is hydroxylated in the liver by the enzyme cytochrome P450 family 2 subfamily R member 1 (CYP2R1) [the primary hepatic hydroxylase, identified by Cheng et al. (19)] to form 25(OH)D (20); Subsequently, 25(OH)D is further hydroxylated in the kidneys by the enzyme cytochrome P450 family 27 subfamily B member 1 (CYP27B1) to produce 1,25-dihydroxyvitamin D [1,25(OH)_2_D], the biologically active form of vitamin D (21, 22).

### 2.2 Vitamin D receptor and its functions

The vitamin D receptor (VDR) is a nuclear receptor that exhibits widespread and crucial expression across a multitude of cell types (23). When VDR binds to 1,25-dihydroxyvitamin D_3_ [1,25(OH)_2_D_3_], it forms a heterodimer with the retinoid X receptor (RXR) (22, 24). This heterodimer subsequently binds to vitamin D response elements (VDREs) to modulate the expression of specific genes (25, 26). Notably, these genes include key players such as bone gla protein (BGLAP) and cathelicidin antimicrobial peptide (CAMP) (27). The regulation of these genes is vital for several essential functions (28). For instance, it promotes bone formation and regeneration, thereby maintaining skeletal health (29). It also plays a role in modulating energy metabolism and insulin sensitivity, which are crucial for metabolic homeostasis (30). Additionally, it enhances host immune defense capabilities, bolstering the body's resistance to infections (31). Collectively, these functions contribute to enhancing overall metabolic health and well-being.

### 2.3 Regulation mechanisms of the vitamin D signaling pathway

The synthesis and degradation of 1,25(OH)_2_D are tightly controlled by a three-armed feedback network (see Figure 1) (32–34). Parathyroid hormone (PTH), fibroblast growth factor-23 (FGF-23) and 1,25(OH)₂D itself jointly dictate the balance between activation and catabolism: low serum calcium or elevated PTH rapidly up-regulates renal CYP27B1 while suppressing cytochrome P450 family 24 subfamily A member 1 (CYP24A1), thereby boosting 1,25(OH)₂D production and intestinal calcium uptake (35, 36); conversely, adequate calcium or rising FGF-23 down-regulates CYP27B1 and induces CYP24A1, converting the hormone to the inactive metabolite 24,25-dihydroxy vitamin D [24,25(OH)_2_D] for urinary excretion (37, 38). Throughout the body, the vitamin-D-binding protein (DBP) acts as a high-affinity shuttle that protects 25(OH)D and 1,25(OH)₂D from rapid clearance and delivers them to target organs (39). This synchronized regulation keeps circulating vitamin D within a narrow physiological window, preventing both deficiency and hypercalcaemic toxicity while ensuring optimal calcaemic and extra-skeletal effects (40, 41).

### 2.4 The interplay of vitamin D signaling with other pathways

In recent years, with the deepening of research into the functions of vitamin D, its roles in immune regulation, cell proliferation and differentiation, and tumorigenesis have gradually been unveiled (42). Particularly noteworthy is the activation of the VDR and its interactions with signaling pathways such as Hedgehog and Wnt/*β*-catenin, which have emerged as hot topics in current research (43). These interactions are of great significance in various physiological and pathological processes.

The Hedgehog signaling pathway is vital for embryonic development and tissue homeostasis, and its abnormal activation is closely related to the occurrence and development of a variety of diseases (44). Studies have shown that vitamin D and its metabolites can regulate cell fate and tissue development through interactions with the Hedgehog signaling pathway (45). For instance, in mouse models, vitamin D, through its active form 1,25(OH)_2_D_3_ binding to the VDR, can inhibit the over-activation of the Hedgehog signaling pathway, thereby reducing the proliferation and migration of tumor cells (46). Moreover, vitamin D also inhibits the expression of target genes in the Wnt/*β*-catenin signaling pathway by activating the deacetylase Sirtuin 1 (SIRT1) (47). This activation promotes the deacetylation and nuclear exclusion of *β*-catenin, thereby reducing cell proliferation and tumor progression. Additionally, 1,25(OH)₂D₃ can also induce the relocalization of *β*-catenin from the cell nucleus to the adherens junctions of the cell membrane, thereby regulating the transmission process of the Wnt/*β*-catenin signaling pathway (48). This interaction has been reported in various cancers, including colorectal cancer and melanoma, and is associated with better prognosis and stronger anti-tumor immune responses (49).

Vitamin D has remarkable anti-inflammatory properties and modulates inflammatory responses via various mechanisms (50). 1,25(OH)₂D suppresses the production of pro-inflammatory cytokines like tumor necrosis factor-α (TNF-α), interleukin-6 (IL-6), and interleukin-1β (IL-1β) (51), and boosts the expression of anti-inflammatory cytokines such as interleukin-10 (IL-10) (52). Moreover, vitamin D can reduce inflammation by regulating the functions of macrophages (53) and dendritic cells (54). This anti-inflammatory effect is significant in the context of inflammatory-related diseases and may contribute to the overall health benefits of vitamin D.

In addition to the major signaling pathways mentioned above, vitamin D also interacts with several other signaling pathways. For example, vitamin D regulates the insulin-like growth factor 1 (IGF-1) signaling pathway, significantly increasing serum IGF-1 levels, especially when the vitamin D dosage is low (≤1,000 IU/day) and the intervention duration is short (≤12 weeks). This interaction may affect cell growth and metabolism by regulating the synthesis and secretion of growth hormone (GH) and IGF-1 (55). Furthermore, vitamin D can reduce the secretion of inflammatory factors by inhibiting the phosphorylation of nuclear factor kappa-B (NF-*κ*B), while simultaneously activating the nuclear factor E2-related factor2 (Nrf2) signaling pathway to enhance the antioxidant capacity of cells. This interaction holds potential therapeutic value in alleviating oxidative stress damage and improving inflammatory-related diseases (56).

**Figure 2:**
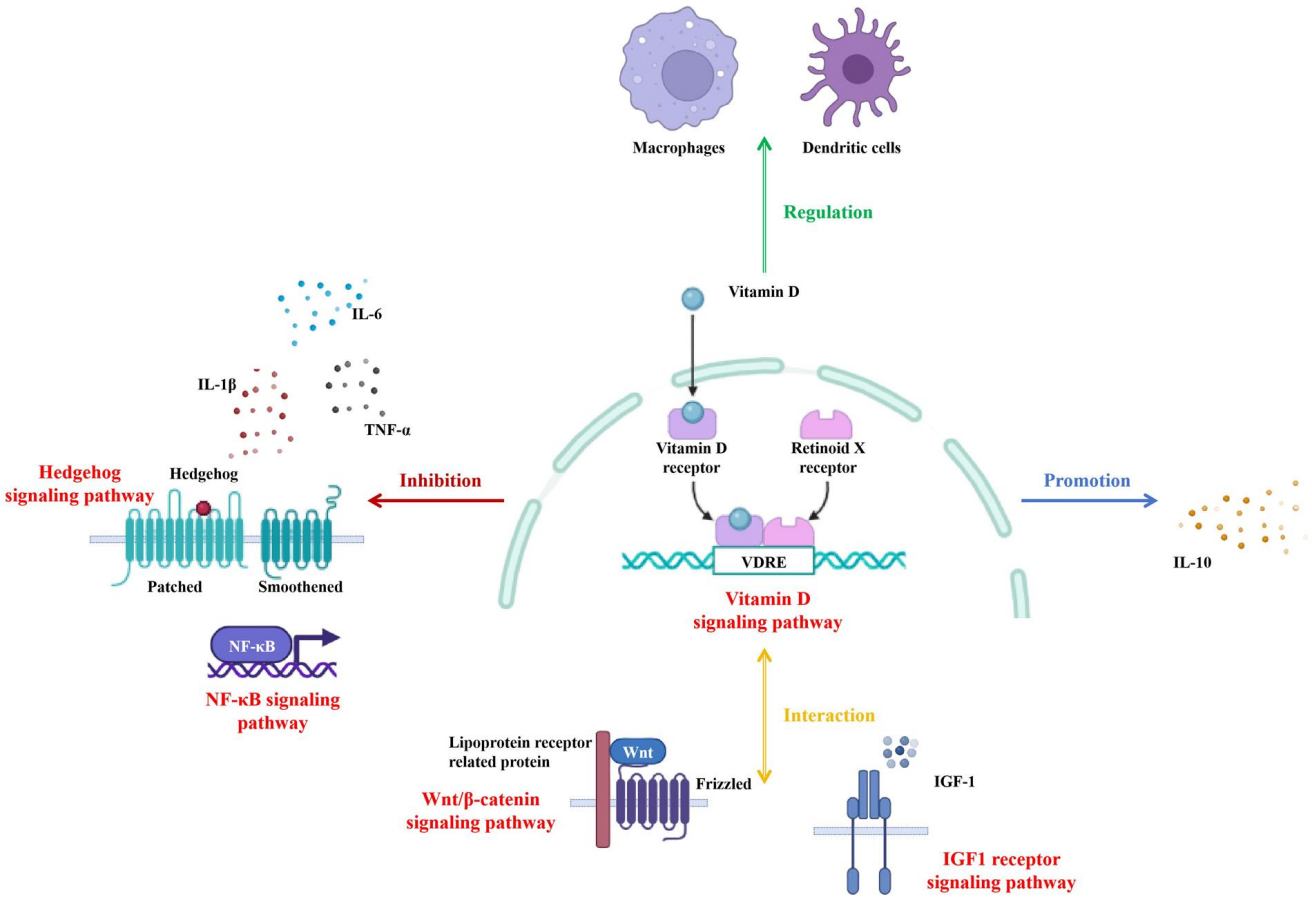
Interaction of vitamin D with other signaling pathways.

In summary, the interactions between the vitamin D signaling pathway and other signaling pathways are of great significance in various physiological and pathological processes (see Figure 2). Further exploration of the molecular mechanisms underlying these interactions will not only enhance our understanding of the biological functions of vitamin D but also provide new strategies for the prevention and treatment of related diseases. Future studies should continue to delve into the specific mechanisms and potential therapeutic applications of these interactions.

## 3 Childhood obesity and vitamin D interaction

### 3.1 The impact of childhood obesity on VDD

Over the past few years, a growing number of studies have unveiled the strong connection between childhood obesity and VDD (57). Obesity is not only a common health issue but also a significant factor that can alter vitamin D status (58). Research by Garfein et al. (59) has shown that changes in body mass index (BMI) from childhood to adolescence are closely associated with VDD, with obese children exhibiting markedly lower levels of serum 25(OH)D. In China, a study comparing 50 obese children with 30 healthy children found that the obese group had significantly lower 25(OH)D levels, and the severity of obesity was inversely correlated with 25(OH)D concentrations (60). These findings highlight the multifaceted nature of the relationship between obesity and vitamin D status, which is further explored through various underlying mechanisms and contributing factors in the following sections.

#### 3.1.1 The volume dilution and fat storage hypothesis

Obese children have a greater amount of adipose tissue, which provides additional storage for vitamin D, potentially leading to reduced serum vitamin D concentrations (61). Here, the “volume dilution” concept, a well-recognized pharmacokinetic phenomenon based on the “volume of distribution (Vd)” principle, refers to the reduction in serum vitamin D concentration caused by an increased total volume of distribution of vitamin D in the body (62). As a fat-soluble vitamin, vitamin D exhibits a high affinity for adipose tissue; thus, in obese children, the expansion of adipose tissue significantly increases the Vd of vitamin D (63).

Data from existing studies indicate that adipose tissue in obese individuals can store large amounts of vitamin D, thereby significantly increasing its distribution volume within the body (64). According to the volume dilution hypothesis, the core necessity of this term lies in its ability to precisely describe the pharmacokinetic mechanism: compared with normal-weight children, obese children have a larger Vd for vitamin D (65). When the total amount of vitamin D synthesized endogenously or acquired exogenously is similar, the same amount of vitamin D must be distributed across a larger bodily volume, resulting in lower serum vitamin D levels (66). This phenomenon is commonly observed in obese populations and may contribute to the increased risk of VDD.

Moreover, obese children typically have a poorer response to vitamin D supplements, further exacerbating the problem of VDD (67). This poor response can also be partially explained by volume dilution: the increased Vd requires a higher dose of vitamin D to reach the same serum concentration as normal-weight children, as studies suggest that obese individuals have lower bioavailability of vitamin D, which may be due to the sequestration of vitamin D by adipose tissue (68). Adipose tissue not only stores vitamin D but may also restrict its release into the bloodstream, making it difficult for supplemented vitamin D to be fully utilized (69). Additionally, obese children may have different metabolic rates and vitamin D metabolic pathways compared to children with normal weight, affecting the efficiency of vitamin D absorption and utilization (70). These factors collectively make obese children more prone to VDD, thereby increasing associated health risks.

#### 3.1.2 MASLD

In the context of childhood obesity, MASLD is not only a common complication related to obesity but may also further worsen VDD, creating a vicious cycle (71). The excessive proliferation and dysfunction of adipose tissue in obese children are crucial factors in the development of MASLD (72). Furthermore, the progression of MASLD can influence vitamin D metabolism and utilization through various mechanisms, exacerbating VDD (73).

Firstly, adipose tissue in obese children is not only the primary site for energy storage but also possesses endocrine functions, secreting a variety of pro-inflammatory cytokines (such as TNF-α and IL-6) and adipokines (such as leptin and adiponectin) (74). These factors can trigger low-grade chronic inflammation, which in turn affects liver metabolic functions and promotes the development of MASLD (75). In the state of MASLD, liver inflammation and lipid metabolism disorders can further reduce the liver's ability to synthesize and activate vitamin D (76). Research indicates that the expression level of VDR in the liver is negatively correlated with the severity of MASLD (77). As MASLD progresses, liver inflammation intensifies, VDR expression levels decrease, and the liver's ability to activate vitamin D is correspondingly weakened, thereby exacerbating VDD (78).

Secondly, adipose tissue itself has a storage and buffering effect on vitamin D (79). Obese children have a larger volume of adipose tissue, making it easier for vitamin D to be stored in fat cells and reducing its concentration in the blood (80). Moreover, the inflammatory state of adipose tissue in individuals with MASLD can also reduce the activity of enzymes related to vitamin D metabolism (such as CYP2R1 and CYP27A1), further decreasing the activation and release of vitamin D, thus forming a vicious cycle of VDD (81).

In summary, in the context of childhood obesity, MASLD is not only a consequence of VDD but can also further intensify it through multiple mechanisms, including inflammation, metabolic disorders, and abnormal adipose tissue function (82). This complex interplay suggests that in the health management of obese children, attention should be paid to both the nutritional status of vitamin D and liver metabolic functions to break this vicious cycle and improve the overall health of children.

#### 3.1.3 Other influencing factors

In the context of childhood obesity, the VDD in obese children is influenced by a multitude of factors (83). Beyond the interplay between obesity itself and MASLD, other elements can also significantly impact vitamin D status (84). For instance, research indicates that the usage rate of vitamin D supplements is lower among overweight or obese students, which may be a key factor contributing to VDD (85). Data from the National Health and Nutrition Examination Survey (NHANES) in the United States reveal that the prevalence of VDD in obese children is significantly higher than in children with normal weight, reaching 49% (86). Additionally, lifestyle behaviors, such as lack of exercise and outdoor activities, can also increase the risk of VDD (87). These behaviors not only reduce sun exposure, thereby affecting the skin's synthesis of vitamin D, but may also lead to unhealthy dietary habits, further decreasing vitamin D intake (88).

Sunlight is the primary source of vitamin D for the human body, with approximately 90% to 100% of vitamin D derived from sun exposure (89). However, in temperate climates during winter and in high latitude regions, the ultraviolet B radiation in sunlight is weaker, significantly reducing the skin's ability to synthesize vitamin D (90). Furthermore, cultural clothing practices, the use of sunscreen, and skin pigmentation can also lower the efficiency of vitamin D synthesis (91). Obese children, due to reduced outdoor activities and insufficient sun exposure, face an increased risk of VDD (92).

Some studies also point out that the dietary habits of obese children often lack foods rich in vitamin D, such as fish, egg yolks, and fortified foods, leading to inadequate vitamin D intake (93). For example, research suggests that obese children may consume less milk because they tend to drink sugary beverages and juices, whereas milk is an important source of vitamin D in children's diets (94). Additionally, breakfast consumption is associated with higher vitamin D levels, possibly due to the fortified vitamin D in breakfast cereals and milk (95).

In conclusion, the VDD in obese children is the result of the combined effects of multiple factors (96). To improve the vitamin D status in obese children, a comprehensive approach should be adopted, including increasing outdoor activity time, the rational use of vitamin D supplements, and improving dietary structure (97). This not only helps prevent and improve MASLD but also holds significant importance for the overall health of obese children.

### 3.2 The impact of VDD on obesity

The influence of VDD on obesity has garnered significant attention, particularly in studies hypothesizing the role of VDD in the pathogenesis of obesity (98). Some researchers suggest that serum 25(OH)D levels depend on the storage of vitamin D in adipose tissue, indicating that adipose tissue may influence obesity by regulating vitamin D levels (99). Shi et al. (100) previously noted that 1,25-(OH)₂D coordinates adipogenesis and lipolysis by modulating calcium signaling in adipocytes. Moreover, 1,25-(OH)₂D can increase lipolysis and isoproterenol-stimulated lipolysis, thereby reducing triglyceride accumulation. Thus, vitamin D intake may decrease intracellular fat accumulation and enhance lipolysis (101). The relationship among VDD, leptin, and obesity has been a focal point in prior research. Leptin, a hormone expressed in adipocytes, circulates in serum in a free form, with its expression and secretion correlating with body fat and adipocyte size (102, 103). An early study highlighted the close association between obesity and leptin, which regulates body fat mass and plays a crucial role in weight control (103). Leptin levels are directly proportional to fat mass. Circulating leptin molecules convey information about energy storage in adipose tissue to the brain (hypothalamus), reducing appetite and influencing energy expenditure. It is proposed that circulating vitamin D participates in leptin synthesis in the body, with its deficiency leading to decreased leptin levels. However, 25(OH)D stored in adipocytes can inhibit leptin secretion (104, 105). Both VDD and increased 25(OH)D content in adipocytes in obese individuals can result in lower leptin levels (106). In obese children with VDD, a state of low leptin levels can occur (107). However, studies on serum leptin have shown that obesity is characterized not by leptin deficiency but by elevated leptin levels (108). Research indicates that there is a threshold level for serum leptin; above this level, leptin cannot enter the cerebrospinal fluid and reach the hypothalamic regions that regulate appetite (109). High serum leptin levels may merely reflect the high adipose tissue levels in the body (110). Thus, the concept of “leptin resistance” in the obese state is considered (111). Based on the relationship between leptin and obesity, studies have found that vitamin D supplementation can enhance the body's utilization of leptin (112). Overall, leptin is involved in fat metabolism, and vitamin D not only participates in leptin synthesis but also counteracts “leptin resistance” in obesity (113). The close relationship among these three factors suggests that vitamin D supplementation may play a role in obesity intervention, with further research needed to elucidate their deeper connections (114). In addition to the aforementioned evidence supporting the impact of vitamin D on obesity, some experiments have found that VDD can lead to increased PTH release, which in turn promotes calcium influx into adipocytes, thereby stimulating adipogenesis (115). Thus, VDD influences obesity through multiple mechanisms and is one of the many risk factors for childhood obesity.

### 3.3 Vitamin D and hypertension in pediatrics

Hypertension has emerged as a growing public health concern in children and adolescents, with long-term implications for adult cardiovascular disease risk. Accumulating evidence suggests a potential link between vitamin D status and blood pressure regulation in the pediatric population, though findings remain partially inconsistent (116).

Epidemiologically, prospective birth cohort studies have demonstrated that low vitamin D status in early life correlates with elevated systolic blood pressure (SBP) during childhood and adolescence. Specifically, persistent low plasma 25(OH)D levels (birth: <11 ng/mL; early childhood: <25 ng/mL) were associated with a 2.04-fold increased risk of SBP ≥75th percentile in children aged 3–18 years (117). However, a systematic review of observational studies noted heterogeneous results, with most non-prospective studies failing to confirm a significant association (118), possibly due to limitations in blood pressure measurement and confounder adjustment.

The underlying mechanisms linking vitamin D to hypertension involve multiple pathways. 1,25(OH)₂D, the active form of vitamin D, functions as a negative regulator of the renin-angiotensin system (RAS)—a key pathway in blood pressure homeostasis (119, 120). VDD may deregulate RAS activity by increasing renin gene expression, leading to elevated angiotensin II levels and subsequent vasoconstriction (121, 122). Additionally, vitamin D contributes to vascular health by promoting endothelial nitric oxide release and reducing vascular inflammation, with deficiency associated with microvascular endothelial dysfunction in young individuals (123).

Clinically, these findings highlight the potential of early vitamin D optimization as a preventive strategy for childhood hypertension. Given the high prevalence of vitamin D insufficiency in pregnant women and children worldwide, screening and targeted supplementation may help mitigate long-term blood pressure risks. However, further well-designed clinical trials are needed to clarify the efficacy of vitamin D supplementation in managing pediatric hypertension and to establish optimal dosage guidelines.

## 4 Correction of VDD in obese children

### 4.1 The impact of vitamin D supplementation on obesity

The impact of vitamin D supplementation on obesity remains a contentious issue, with ongoing debate surrounding its efficacy in managing this widespread health concern. A growing body of evidence from animal studies suggests that vitamin D supplementation may confer beneficial effects on obesity (124). For instance, Cordeiro et al. (125) demonstrated that vitamin D supplementation in a high-fat diet-induced obese rat model significantly reduced the rate of weight gain, improved abdominal fat deposition, and optimized lipid distribution. These findings suggest that vitamin D supplementation could be a promising strategy for mitigating weight gain and combating obesity. Consistent with these observations, Sergeev et al. (126) reported that high vitamin D_3_ intake in high-fat diet-induced obese mice led to a reduction in white adipose tissue weight, improved obesity-related biomarkers, and induced apoptosis in adipocytes via Ca^2+^-dependent pathways, thereby reducing adipose tissue accumulation.

Conversely, several studies have failed to establish a significant impact of vitamin D supplementation on obesity (127). A comprehensive meta-analysis, which synthesized data from numerous intervention studies, concluded that there is no substantial evidence to support the notion that vitamin D supplementation confers benefits on body weight (128). Similarly, Abbas (65) highlighted that while vitamin D supplementation may help prevent the onset of obesity, it does not necessarily result in weight loss. In addition, studies in animal models of MASLD have shown that although vitamin D supplementation can ameliorate hepatic inflammation and insulin resistance, its effects on body weight are negligible (129).

In summary, while some animal studies indicate that vitamin D supplementation may have a positive impact on obesity, the overall findings are inconsistent. The causal relationship between vitamin D supplementation and obesity treatment remains to be elucidated. Future research should focus on determining the optimal dosage and delivery methods of vitamin D supplementation, as well as exploring the underlying mechanisms in various obesity models, to better understand its potential role in obesity management.

### 4.2 Vitamin D supplementation for the treatment of obesity in children

VDD is highly prevalent among obese children and represents a significant clinical challenge. The management strategies for VDD in this population vary significantly across different countries. The guidelines published by the Endocrine Society in 2011, which have been widely adopted, recommend that obese children receive at least 2–3 times the standard dose of vitamin D (a minimum of 6,000–10,000 IU/day) to achieve serum 25(OH)D levels above 30 ng/mL, followed by a maintenance dose of 3,000–6,000 IU/day (130). The Nutrition Committee of the French Pediatric Society suggests that children at risk of overweight or obesity could benefit from vitamin D supplementation during winter, with single doses of 80,000 or 100,000 IU for 5- to 10-year-old obese children, or continuous supplementation within the 1- to 10-year age range (129). For obese children with VDD, high-dose treatment is generally recommended by guidelines.

Obese children have been shown to have a reduced response to vitamin D supplementation. Tayde et al. (131) demonstrated that after a single dose of 150,000 IU of vitamin D, the increase in serum vitamin D concentration in obese children was 2.2 times lower than that in normal-weight children. Similarly, a non-randomized trial indicated that obese children had a significantly lower response to standard-dose vitamin D_3_ compared to non-obese children (132). These findings underscore the need for higher doses of vitamin D supplementation in obese children to effectively address VDD. For instance, a study in China provided weekly supplementation of 25,000 IU of vitamin D to obese children aged 8–18 years with vitamin D insufficiency or deficiency, resulting in 85% of the children achieving adequate vitamin D levels (133). In another randomized controlled trial, 35 obese adolescents with VDD received 4,000 IU/day (28,000 IU/week) of vitamin D supplementation, and after 6 months, 93% of the participants reached sufficient levels (134). Asghari et al. (135) conducted a group-controlled trial involving 378 children and found that 25(OH)D concentrations significantly increased with daily doses of 1,000 and 2,000 IU of vitamin D, whereas no significant change was observed in the 600 IU/day group.

The potential cardiovascular benefits of vitamin D supplements in obese children have also garnered attention. A randomized clinical trial showed that vitamin D_3_ supplementation improved in plasma glucose in obese children but did not significantly affect on lipid profile, insulin resistance markers, or inflammation (136). Another study found that monthly supplementation with 100,000 IU of vitamin D_3_ increased 25(OH)D levels in obese adolescents without significantly impacting endothelial function (137). These findings suggest that while vitamin D supplements may positively influence certain metabolic indicators in obese children, their comprehensive impact on cardiovascular health requires further investigation.

Clinical trials have provided valuable insights into the effects of vitamin D supplementation in obese children. A randomized, double-blind, controlled trial involving 225 obese children aged 10–18 years evaluated the impact of vitamin D_3_ supplements (1,000 IU/day and 2,000 IU/day) compared to 600 IU/day on various health parameters. The results indicated a significant increase in serum 25(OH)D levels, with good tolerability and no incidence of hypercalcemia. After 6 months, the 1,000 IU/day group experienced significant reductions in central systolic, central diastolic, and systemic diastolic blood pressures. The 2,000 IU/day group exhibited significant improvements in insulin sensitivity at 3 and 6 months and a significant decrease in fasting blood glucose concentration at 6 months. However, no significant differences were observed in arterial endothelial function, arterial stiffness, systemic inflammation, or lipid profiles among the groups (138).

Another randomized, double-blind, controlled trial in 152 obese adolescents assessed the effects of vitamin D supplements (1,200 IU/day) on weight loss programs. After 26 weeks, the vitamin D group showed a significant increase in serum 25(OH)D levels, while the placebo group did not. No significant differences were observed between the groups in BMI, BMI percentile, fat mass, muscle mass, or bone density. The study concluded that vitamin D supplementation did not significantly impact weight loss or cardiovascular risk factors (139).

A randomized, double-blind, controlled trial in 29 obese African-American adolescents evaluated the effects of weekly 50,000 IU vitamin D_2_ supplementation on 25(OH)D levels and insulin homeostasis. After 12 weeks, the vitamin D treatment group exhibited a significant increase in serum 25(OH)D levels, whereas the placebo group did not. Despite the increase in vitamin D levels, no significant differences were observed in insulin secretion or sensitivity between the groups. However, in the vitamin D treatment group, serum 25(OH)D levels were positively correlated with fasting insulin and high-density lipoprotein (HDL) levels (140).

In other regions, a randomized, double-blind, controlled trial in 62 Korean children assessed the effects of vitamin D_3_ supplements (2,000 IU/day) on vitamin D-deficient [25(OH)D < 20 ng/mL] and obese children. After 8 weeks, 61.9% of normal-weight children achieved adequate vitamin D levels [25(OH)D ≥ 30 ng/mL], compared to 47.6% of obese children. Vitamin D supplementation significantly increased serum 25(OH)D levels but did not significantly affect insulin sensitivity or lipid levels (141). Another randomized, double-blind, controlled trial in 96 obese children in Sri Lanka evaluated the effects of weekly 50,000 IU vitamin D_2_ treatment, 2,500 IU vitamin D_2_ supplementation, and placebo. The treatment group showed significant improvements in BMI, waist circumference, body fat percentage, serum calcium, vitamin D levels, and alanine aminotransferase (ALT) levels, while the placebo group did not (142).

Across the 12 randomised trials summarised in Table 1, vitamin D regimens ranged from 600 IU/day to 50,000 IU/week, follow-up from 1 to 26 weeks, and baseline 25(OH)D from 8 to 25 ng/mL; participants' age, ethnicity, season of enrolment and concomitant lifestyle counselling were rarely uniform. Only five studies were double-blind and placebo-controlled; sample sizes were generally <100, giving a high risk of type II error. Notably, meta-analytic outcomes for BMI or fat mass showed no significant change (*p* > 0.05 in pooled random-effects), whereas improvements in HOMA-IR or systolic BP were confined to sub-groups with baseline 25(OH)D < 20 ng/mL and IL-6 < 3 pg/mL. These heterogeneities preclude definitive dosing recommendations and underscore the need for larger, longer, and individually stratified trials that standardise vitamin D status entry criteria, inflammatory co-interventions, and outcomes reporting.

**Table 1:** Characteristics of 12 studies on vitamin D supplementation in obese children.

Region	Country	Sample Size (n)	Age Range (years)	Follow-up Period	Treatment Doses	Results	References
Asia	India	44	5–12	1 month	Single dose of 150,000 IU	2.2-fold lower serum 25(OH)D rise in obese children	Tayde et al. (131)
	Iran	90	2–14	6 weeks	50,000 IU/week	Poor therapeutic response in obese group	Motlaghzadeh et al. (132)
	Iran	378	6–13	6 or 12 months	600; 1,000; 2,000 IU/days	Significant increase in 25(OH)D concentrations with 1,000 and 2,000 IU doses; no significant change in the 600 IU/day group	Asghari et al. (135)
	Korea	62	7–13	8 weeks	2,000 IU/day	Increased serum 25(OH)D levels with vitamin D treatment; no effects on insulin sensitivity or lipid levels	Chung et al. (141)
	Sri Lanka	96	5–15	24 weeks	25,000; 50,000 IU/week	Significant improvements in BMI, waist circumference, body fat percentage, serum calcium, vitamin D levels, and ALT levels with high-dose vitamin D	Samaranayake et al. (142)
Europe	The Netherlands	109	8–18	9 weeks	25,000 IU/week	High-dose vitamin D effective, well-tolerated in obese children	Radhakishun et al. (133)
	Poland	152	6–14	26 weeks	1,200 IU/day	Significant serum 25(OH)D increase with vitamin D; no impact on weight or cardiovascular risk factors	Brzeziński et al. (139)
North America	USA	35	9–19	6 months	4,000 IU/day	Increased 25(OH)D fasting insulin, HOMA-IR, and leptin-to-adiponectin ratio in vitamin D group	Belenchia et al. (134)
	USA	58	12–18	12 weeks	2,000 IU/day	Improved plasma glucose with 2,000 IU dose; no impact on lipid profile, insulin resistance markers, or inflammation	Nader et al. (136)
	USA	19	13–18	3 months	100,000 IU/month	Increased 25(OH)D levels and decreased serum PTH in obese adolescents; no impact on flow-mediated dilatation	Javed et al. (137)
	USA	225	10–18	3 or 6 months	600; 1,000; 2,000 IU/days	Reduced blood pressure and fasting glucose concentration with vitamin D; no effect on arterial endothelial function, stiffness, systemic inflammation, or lipid profile	Rajakumar et al. (138)
	USA	29	13–17	12 weeks	50,000 IU/week	Significant serum 25(OH)D increase with vitamin D; no differences in insulin secretion or sensitivity	Sethuraman et al. (140)

IU, international unit; HOMA-IR, homeostasis model assessment of insulin resistance; 25(OH)D, 25-hydroxyvitamin D; BMI, body mass index; ALT, alanine aminotransferase; PTH, parathyroid hormone.

### 4.3 Safety and potential side effects of high-dose vitamin D supplementation

While high-dose vitamin D supplementation is recommended for correcting VDD in obese children, attention to its safety and potential side effects—especially with long-term use—is essential. Vitamin D toxicity is primarily mediated by excessive accumulation of 1,25(OH)₂D, leading to abnormal calcium metabolism, but such cases remain rare when doses are within clinical guidelines.

#### 4.3.1 Defined safety thresholds

The Endocrine Society has established age-specific upper limits for vitamin D intake to avoid toxicity: infants <6 months: ≤1,000 IU/day; 6 months-1 year: ≤1,500 IU/day; 1–3 years: ≤2,500 IU/day; 4–8 years: ≤3,000 IU/day; and ≥8 years: ≤4,000 IU/day for unsupervised supplementation. For therapeutic purposes, supervised doses up to 10,000 IU/day for 8–12 weeks are considered safe in obese children, as supported by clinical trials showing no incidence of hypercalcemia or other adverse events (65, 143).

#### 4.3.2 Short-Term potential Side effects

The short-term side effects of high-dose vitamin D supplementation are predominantly linked to abnormal calcium metabolism, yet clinical evidence consistently confirms these effects are rare, mild, and mostly reversible in otherwise healthy obese children. A large observational study of 22,214 healthy volunteers (with vitamin D supplementation doses ranging from 0 to 55,000 IU/day) demonstrated that serum calcium levels increased by only 0.001 mmol/L per 1,000 IU increment in vitamin D intake, with no statistically significant association between supplementation dose and the risk of hypercalcemia—among 10,940 serum calcium assessments in this cohort, only 1.7% had albumin-corrected calcium levels exceeding 2.6 mmol/L, and these cases were primarily associated with older age or female gender rather than vitamin D dosage (144). Further support comes from a phase 2 trial involving 40 patients receiving 10,000 IU/day of vitamin D_3_ for 4 months, where 2 cases developed hypercalcemia; subsequent evaluations revealed these two patients had undiagnosed primary hyperparathyroidism, indicating the side effect stemmed from pre-existing endocrine dysfunction rather than direct vitamin D toxicity (145).

Hypercalciuria (defined as a urinary calcium/creatinine ratio >0.21) is slightly more common than hypercalcemia, observed in 5.7% of children on long-term high-dose supplementation, but this condition is typically mild and resolves with dose adjustment or temporary discontinuation (64). When clinical symptoms do occur, they are mostly mild and gastrointestinal in nature—including nausea, constipation, and abdominal discomfort—with a meta-analysis of trials in obese children showing that mild gastrointestinal discomfort was reported in only 3.2% of participants receiving doses >5,000 IU/day (138); other potential symptoms such as polyuria, polydipsia, or fatigue are even rarer, with no reports of severe or persistent manifestations in pediatric-specific studies (131). Notably, a phase 2 trial in adults with cancer also reported no severe short-term toxicity, further reinforcing the safety of high-dose supplementation in short-term use (146).

It is important to emphasize that the risk of short-term side effects is not elevated by vitamin D dose alone but is modulated by underlying health conditions: children with pre-existing hyperparathyroidism, renal impairment, or MASLD may be more susceptible to calcium metabolism disturbances, but these groups are not representative of the general obese pediatric population. For otherwise healthy obese children, short-term high-dose vitamin D supplementation has shown no increased risk of clinically significant side effects (144, 146). In summary, short-term high-dose vitamin D supplementation is well-tolerated in obese children; the rare potential side effects are mild and reversible, and they are strongly associated with pre-existing metabolic or endocrine conditions rather than vitamin D itself. Routine monitoring of serum calcium is unnecessary for healthy obese children undergoing short-term high-dose supplementation but is recommended for those with pre-existing renal or parathyroid disorders to ensure safety.

#### 4.3.3 Long-Term potential risks

Long-term use of high-dose vitamin D (exceeding 4,000 IU per day for more than 1 year) may bring additional health risks, most of which are associated with persistent abnormalities in calcium metabolism and potential organ damage. Vascular calcification stands out as a critical concern. Excessive vitamin D can disrupt the balance of calcium deposition in blood vessels, promoting calcium accumulation in the vascular wall (147). This risk is particularly heightened in children with underlying metabolic disorders such as MASLD or hypertension, as their vascular endothelial function is already more vulnerable to disturbances. While direct clinical evidence in pediatric populations remains limited, animal studies have confirmed that sustained high vitamin D levels can trigger vascular smooth muscle cell calcification (148).

Meanwhile, there is a positive correlation between prolonged serum 25(OH)D levels above 50 ng/mL and increased arterial stiffness—a key marker of early vascular damage. A randomized controlled trial focusing on systemic lupus erythematosus (SLE) patients further supported this link, noting that although vitamin D supplementation improved carotid intima-media thickness (IMT) in vitamin D-insufficient participants, those with supraphysiological 25(OH)D levels had a trend toward increased IMT, suggesting a potential “dose-dependent” risk of vascular remodeling (149).

Another notable long-term risk is increased renal burden. Chronic hypercalciuria, a common consequence of long-term high-dose vitamin D supplementation, can elevate the risk of nephrolithiasis by increasing calcium excretion in urine (150). Over time, persistent high calcium concentrations in the renal tubules may also cause subtle tubular damage, especially in children with pre-existing reduced renal function, as their kidneys are less capable of regulating calcium excretion. A systematic review and meta-analysis of 32 randomized controlled trials (involving 23,228 participants) found increased circulating 1,25(OH)₂D is associated with urinary stones and a higher level of circulating 25(OH)D is significantly associated with hypercalciuria urolithiasis (151).

#### 4.3.4 Clinical monitoring recommendations

To mitigate potential risks during high-dose vitamin D supplementation in obese children, standardized regular monitoring protocols are essential in clinical practice. Specifically, baseline and follow-up measurements of serum 25(OH)D, calcium, phosphorus, and creatinine levels should be completed before initiating high-dose supplementation and 8–12 weeks after supplementation. This monitoring interval aligns with the metabolic cycle of vitamin D in the body and enables timely detection of early signs of abnormal calcium metabolism. For children receiving a maintenance dose exceeding 3,000 IU per day, the urinary calcium/creatinine ratio should be evaluated every 6 months, as prolonged high-dose intake may lead to sustained elevation of urinary calcium excretion, thereby facilitating early identification of hypercalciuria risk. Once serum 25(OH)D levels exceed 50 ng/mL or hypercalcemia (serum calcium >10.5 mg/dL) is detected, supplementation should be immediately suspended or the dose reduced to avoid subsequent risks such as vascular calcification and renal injury caused by vitamin D excess. In general, high-dose vitamin D supplementation is safe for obese children under the guidance of clinical guidelines and standardized monitoring; its potential side effects are mostly mild and reversible. However, long-term administration requires careful assessment of metabolic indicators and renal function to balance efficacy and safety.

In summary, high-dose vitamin D supplementation is generally recommended for treating VDD in obese children. However, the heterogeneity of existing studies, including differences in nationality, season, sunlight exposure, and dosing regimens, highlights the need for more large-scale clinical research to accurately quantify and evaluate the optimal dosages for therapeutic interventions. Future studies should aim to address these gaps and provide clearer guidelines for the management of VDD in obese children.

## 5 Conclusion

Childhood obesity and VDD no longer represent two isolated nutritional disorders; rather, they form a self-amplifying cycle that begins in early life and extends far beyond skeletal health. The present review integrates recent clinical, translational and Mendelian-randomisation data to propose three paradigm shifts that should guide the next decade of research and public-health action (1): From “associations” to “feed-forward loops”. Obesity promotes VDD through volume-dilution, adipose sequestration and hepatic MASLD-related hydroxylase dysfunction; conversely (61, 72), VDD aggravates adipogenesis, leptin resistance and low-grade inflammation, locking the child into a feed-forward loop that is difficult to break by lifestyle intervention alone (100, 115). (2) From “one-size-fits-all” to “precision supplementation”. Obese children require 2- to 3-fold higher vitamin D doses to reach repletion, yet pharmacokinetic modelling shows that the incremental benefit plateaus beyond 4,000 IU/day and may be offset by a compensatory rise of CYP24A1-mediated catabolism (130, 131). Genotype–phenotype analyses explain up to 32% of inter-individual variability in 25(OH)D response and should be incorporated into future algorithms for precision dosing (126, 135). (3) From “bone-centric” to “metabolism-centric” end-points. Meta-analytic evidence indicates that vitamin D supplementation in obese youth improves insulin sensitivity and lowers systolic BP, but fails to reduce BMI or fat mass (134, 138). Importantly, these metabolic benefits appear only when baseline 25(OH)D < 20 ng/mL and are lost when inflammation is present, suggesting that adjunctive anti-inflammatory strategies may be required to unlock the full therapeutic potential of vitamin D (139).

Critical knowledge gaps remain: (1) the threshold of adipose-tissue 25(OH)D storage at which hepatic CYP27B1 becomes substrate-limited; (2) the epigenetic regulation of VDR methylation in obese vs. lean children; and (3) the long-term safety of intermittent high-dose boluses (>100,000 IU) on vascular calcification. Multi-omic longitudinal cohorts that integrate metabolomics, epigenomics and gut-microbiome data are urgently needed to move from empirical megadosing to mechanism-driven, personalised supplementation.

Until such evidence is available, we recommend a pragmatic “3-A” strategy for clinicians: Assess: 25(OH)D, IL-6, VDR genotype and MASLD status; Adjust: initial 6,000–10,000 IU/day for 8–12 weeks, then taper to 2,000–3,000 IU/day; Augment: combine vitamin D with anti-inflammatory dietary components and structured outdoor activity to disrupt the obesity-VDD cycle.

By reframing childhood obesity-associated VDD as a systems-level, personalised challenge rather than a simple nutrient deficit, future interventions can be better targeted, more effective and ultimately more sustainable.
